# An Englishman's Account of Some American Hospitals

**Published:** 1902-02-01

**Authors:** 


### AN ENGLISHMAN'S ACCOUNT OF SOME AMERICAN HOSPITALS.

Dr. G. B. Ferguson,[1] President of the British Medical Association, who has lately visited Canada and the United States, has been giving to the Gloucester Branch of the Association some of his experiences in regard to American Hospitals. He says that the nurses are treated with much deference and were not called nurse. "Miss C., may I trouble you for another ligature ?" was the mode in which they were addressed.

Women nurses are the most numerous, though male orderlies are also kept for the bed-pan and urinal work of men. Otherwise men are attended by women nurses. The whole of the flat roof of the Presbyterian Hospital is used as a "solarium," and hammocks, seats, and couches were all about. Here, we think, is a hint that might well be taken by British architects. After 7 p.m. the roof belongs to the nurses. They play games there, and no male person is allowed to interfere or even to look on. Nevertheless they are said to enjoy themselves very much.

The anæsthetic was always ether, poured liberally into a simple cone covered with waterproofing. Children were given the ether on simple lint.

Dr. Ferguson was much impressed with the post-mortem room at the Victoria Hospital, Montreal, where the pathological department is under the care of Professor Adami. Each dead body was kept in its own refrigerating chamber until wanted, when it was brought up in a lift to the post-mortem room, which was thus kept free from all odour of decomposition. "We have really much to learn from the Canadians and Americans as to the best arrangements of a dead-house."

The great character of the American hospitals is the worship of asepsis. So particular are they at many of them that even an assistant surgeon of the hospital may not enter the operating space unless he, too, is cleansed and dressed, and you are warned by a notice in some hospitals not even to touch the brass bar that separates the aseptic from the septic. Adjoining the operating theatre in most hospitals baths and lavatory for the surgeons are found, and it is usual for surgeons to take a bath before an operation and a shower bath afterwards. The surgeons and all engaged in the operation wear canvas boots, linen pants and coats, and a gauze night cap, all sterilised.

At the Johns Hopkins Hospital he noticed the use of silver foil as a dressing after Halsted's operation. This was applied next the skin. "The foil and the ensheathing papers are both sterilised, and many layers of both are used. Then comes a plain gauze dressing, and that is all." Of course, he says, the silver foil forms antiseptic salts with the serum. In all American hospitals the sterilisers of clothing and dressings are of vast size and look like great brass boilers. They are usually supplied with steam from the basement and are used at a pressure of two atmospheres for at least twenty minutes. All water for all purposes is sterilised and filtered. At the Pennsylvanian Hospital there were two cases of gun-shot wound of the abdomen, in both of which all the intestines had been turned out. In one case two wounds had been found and sutured, and in the other three, and both were doing well, which shows how thoroughly the principles of asepsis are understood and practised. It is evident that asepsis of the most complete and thorough character is the keynote to much of the success of American surgery.

[1] Brit. Med. Journ. Jan. 25.

